# Integrated Desalination, Phycoremediation, and Biodiesel Production from Halophilic Microalgae Using Aquaculture Wastewater

**DOI:** 10.3390/biology15070584

**Published:** 2026-04-06

**Authors:** Adel W. Almutairi

**Affiliations:** Biological Sciences Department, Faculty of Science & Arts, King Abdulaziz University, Rabigh 21911, Saudi Arabia; aalmutairi@kau.edu.sa

**Keywords:** *Dunaliella salina*, green energy, microalgae cultivation, phycoremediation, sustainability

## Abstract

Aquaculture wastewater (AWW) contains elevated concentrations of nitrogen, phosphorus, and salts, in addition to many micropollutants that may cause environmental pollution if discharged untreated. This study evaluated the potential of the halophilic microalga *Dunaliella salina* for simultaneous phycoremediation of AWW and production of biodiesel-oriented biomass. Microalgal growth and biochemical composition were compared between AWW and synthetic f/2 medium under controlled laboratory conditions. Results showed that AWW supported efficient microalgal growth, showing a biomass yield of 1.32 g L^−1^ with a productivity of 0.09 g L^−1^ d^−1^, representing 40.88% and 18.42%, respectively, over that obtained in f/2 medium. Cultivation in wastewater also enhanced the volumetric productivity of lipids, proteins, and carbohydrates by 26.20%, 12.46%, and 26.38%, respectively. Significant nutrient removal from AWW was achieved, with high reduction efficiencies for nitrate, nitrite, ammonium, phosphate, and sulfate within the range 76.80–94.10%, along with a decrease in salinity by 29.70%. The lipid fraction was dominated by fatty acid methyl esters suitable for biodiesel production, representing 94.10% of the total lipids. Biodiesel properties met the international fuel standards and were even improved when the microalga was cultivated in AWW. These findings demonstrate that AWW can serve as an effective culture medium for halophilic microalgae, enabling simultaneous wastewater treatment and sustainable biofuel feedstock production.

## Simple Summary

Aquaculture wastewater contains high levels of nutrients and salts, as well as high levels of micropollutants, that can cause environmental pollution if released untreated. This study evaluated the halophilic microalga *Dunaliella salina* for treating aquaculture wastewater while producing biomass for biodiesel. The microalga grew well in wastewater and produced more biomass and valuable compounds compared to synthetic medium. It also removed most nutrients and reduced salinity. The produced lipids were rich in fatty acids suitable for biodiesel production. The results show that aquaculture wastewater can be effectively used to cultivate microalgae for wastewater treatment and sustainable biofuel production.

## 1. Introduction

The rapid expansion of global aquaculture has played a major role in increasing the supply of aquatic food products and supporting global food security. It is reported that aquaculture is presently among the most rapidly expanding sectors in global food production [1]. However, these increasing aquaculture practices have resulted in the generation of large volumes of nutrient-rich wastewater that may cause significant environmental problems if discharged without adequate treatment. Aquaculture wastewater (AWW) contains high concentrations of suspended solids, organic matter, nitrogen compounds, phosphorus, and dissolved salts produced from uneaten feed, fish excreta, and metabolic byproducts [1,2]. The discharge of untreated aquaculture effluents into water bodies can contribute to oxygen depletion, eutrophication, harmful algal blooms, and the overall degradation of aquatic ecosystems [3]. Conventional methods used to treat AWW include sedimentation ponds, biological filtration, mechanical filtration, and chemical treatment processes. Although these technologies can partially reduce pollutant loads, they often require high energy inputs, significant operational costs, and continuous maintenance [4]. Moreover, most conventional approaches focus primarily on pollutant removal without generating valuable by-products. Therefore, sustainable and economically viable alternatives are needed to improve the environmental performance of aquaculture systems.

Microalgae-based wastewater treatment has emerged as an effective and environmentally friendly technology to overcome these challenges. Microalgae are photosynthetic microorganisms capable of converting carbon dioxide using solar energy into organic biomass while assimilating nutrients from their environment in a process known as phycoremediation [5]. Compared with conventional wastewater treatment technologies, phycoremediation offers numerous advantages, such as reduced sludge production, recovery of valuable resources, and lower energy requirements.

One of the major challenges in industrial microalgae cultivation is the high cost associated with synthetic culture media. Commonly used synthetic media, such as f/2 medium, contain several nutrients and trace elements required for optimal microalgal growth, which increases production costs when applied at industrial scale [6]. Microalgae are widely recognized for their strong capacity to eliminate inorganic nutrients from wastewater. During cultivation, they take up nitrogen, primarily as nitrate, nitrite, or ammonium, and phosphorus as phosphate. These nutrients are incorporated into cellular constituents, including proteins, nucleic acids, and phospholipids, which are essential for cellular growth and metabolism [7]. In addition to nutrient removal, microalgae contribute to carbon dioxide mitigation through photosynthetic carbon fixation, which further enhances the environmental benefits of this process [8]. Previous studies have reported nutrient removal efficiencies exceeding 70–90% in microalgae-based treatment systems under appropriate cultivation conditions [5,9]. Consequently, the use of wastewater as an alternative nutrient source has attracted considerable attention. AWW is particularly suitable for microalgal cultivation because of its relatively high concentrations of dissolved nutrients and moderate organic load [1]. In many aquaculture systems, wastewater also contains elevated salinity levels, especially in marine or brackish water operations with salinity range 3–33 ppt [10]. Salinity conditions as high as 15 ppt may limit the growth and proliferation of freshwater microalgae [11,12], but can favor the cultivation of halotolerant or halophilic microalgal species [13]. Integrating marine microalgae cultivation into AWW treatment systems therefore provide an opportunity to recycle nutrients and improve water quality while producing valuable biomass.

Microalgae have gained considerable attention as a sustainable raw material for biofuel generation. Numerous species can accumulate substantial amounts of lipids within their cells, and these lipids can be transformed into biodiesel via transesterification processes [14,15]. In comparison with conventional oil crops, microalgae present several benefits, such as superior photosynthetic efficiency, faster growth, and the ability to be cultivated on non-arable land using saline water or wastewater resources [16]. Furthermore, microalgal cultivation does not directly compete with agricultural land used for food production, supporting its potential as a more sustainable source of biofuel feedstock. Biodiesel produced from microalgal lipids is mainly composed of fatty acid methyl esters (FAMEs), which exhibit desirable fuel characteristics, including a high cetane number and strong biodegradability [17]. The overall quality of biodiesel largely depends on the fatty acid composition of the original lipid source. Consequently, assessing the biochemical composition and fatty acid profiles of microalgal biomass grown under varying cultivation conditions is important to evaluate its potential for biodiesel applications.

Among the different microalgal species explored for applications in wastewater remediation and biofuel production, the halophilic green microalga *Dunaliella salina* has received increasing attention [18,19,20]. This unicellular microalga is distinguished by its extraordinary tolerance to high salinity and its ability to thrive across a broad range of saline environments, from seawater to nearly saturated salt solutions [13,21]. Beyond its salinity resilience, *D. salina* is known for synthesizing a variety of valuable compounds, including carotenoids, particularly β-carotene, as well as glycerol, proteins, and lipids [21,22]. The commercial production of β-carotene from this microalga is already established in the market, where it is utilized in food, pharmaceuticals, and cosmetics. In recent years, *D. salina* has also been investigated as a promising biofuel feedstock because it can accumulate significant lipid reserves under stress conditions such as nutrient deprivation or elevated salinity [23,24]. Applying *D. salina* in AWW treatment presents further benefits due to its capacity to adapt to high salinities typically encountered in marine aquaculture systems in hyper-arid nations like Saudi Arabia. Since wastewater generated by many aquaculture operations often contains relatively high salt concentrations (typically in the range of 15–40 ppt, depending on the culture system and species) [25,26], halophilic microalgae like *D. salina* can be cultivated efficiently without extensive dilution. Moreover, integrating microalgal growth into wastewater treatment processes can lower nutrient levels and enhance water quality prior to discharge or reuse.

Despite the promising potential of microalgae-based wastewater treatment systems, many challenges remain to be considered for industrial implementation. Environmental factors such as temperature, light intensity, nutrient availability, and microbial contamination can significantly influence microalgal growth and biochemical composition [27]. Therefore, detailed studies evaluating the growth performance, nutrient removal efficiency, and biochemical characteristics of microalgae cultivated in specific wastewater environments are essential. In this context, AWW represents a valuable resource for supporting microalgal cultivation while simultaneously reducing environmental pollution. Integrating microalgae systems with AWW treatment may contribute to the development of sustainable circular bioeconomy schemes in which waste streams are converted into valuable resources. Moreover, using the produced biomass for biodiesel production or other value-added products may enhance the economic feasibility of such integrated systems. Therefore, the present study investigates the potential of *D. salina* for the phycoremediation of AWW while simultaneously producing biomass suitable for biodiesel production. Specifically, this work aims to evaluate the growth performance of *D. salina* cultivated in AWW compared with f/2 as a standard synthetic growth medium, assess nutrient removal efficiencies, determine the biochemical composition of the produced biomass, and analyze FAMEs profiles of the extracted lipids. By combining wastewater treatment with biomass valorization, this study contributes to developing a sustainable strategy for environmental management and renewable bioenergy production.

## 2. Materials and Methods

### 2.1. Aquaculture Wastewater

AWW was collected from the National Aquaculture Group (NAQUA), Al Lith, KSA. Samples were transported to the laboratory, filtered through a 5 µm plankton net and stored at 4 °C until further use and analysis. Physicochemical parameters of AWW, including nitrate, nitrite, ammonium, sulfate, and phosphate, were determined using standard spectrophotometric methods following APHA protocols [28]. Nitrate was measured using the UV spectrophotometric method, nitrite by the sulfanilamide method, ammonium using the indophenol blue method, and phosphate by the ascorbic acid method. Sulfate concentration was determined using the turbidimetric method. pH was measured using a calibrated digital pH meter, while salinity was determined using a conductivity-based salinity meter. The main measured characteristics of the AWW and calculated values of f/2 are presented in Table 1.

### 2.2. Microalgal Cultivation

The marine microalga *Dunaliella salina* was provided by the New Energy and Environmental Laboratory (NEEL) at Chengdu University, Chengdu, China, which was originally isolated from hypersaline water with an average salinity of 45.3 ppt [13]. Stock axenic cultures were maintained under laboratory conditions using 50 mL of standard f/2 medium [29]. For experimental cultivation, cells were transferred into glass photobioreactors as previously described [30], each containing 1.6 L of either f/2 medium or AWW. All cultures were inoculated to reach an initial optical density of 0.05 and incubated for 18 days at 25 °C under a light intensity of 100 μmol photons m^−2^ s^−1^ with 18:6 h light:dark cycle. Continuous aeration was provided from the bottom of the reactors throughout the cultivation period to ensure proper mixing and CO_2_ supply.

### 2.3. Growth Monitoring and Biomass Determination

Algal growth was monitored by measuring the optical density at 680 nm (OD_680_). For dry biomass determination, 5–10 mL of the culture was filtered through a pre-weighed 0.45 µm membrane filter and subsequently dried at 80 °C until a constant weight. Biomass productivity (as g L^−1^ d^−1^) was calculated from the dry biomass values at the early and late exponential phase as previously described [31]. Desalination efficiency (%) was calculated from the reduction in salinity relative to the initial value.

### 2.4. Biochemical Composition Analysis

Total lipids were extracted using a mixture of chloroform:methanol (2:1, *v*/*v*) following the method of Bligh and Dyer [32] and quantified gravimetrically. For biodiesel preparation as FAMEs, the harvested biomass was processed following a modified protocol from Christie [33] as adapted by Abomohra et al. [34]. Prior to lipid extraction, 40 μg of 1,2,3-trinonadecanoylglycerol was added as internal standard. Carbohydrate and protein contents were determined using standard analytical procedures [35,36]. In addition, productivities of macromolecules (mg L^−1^ d^−1^) were calculated at the end of the exponential phase from biomass productivity and corresponding cellular contents.

### 2.5. Fatty Acid Analysis and Biodiesel Properties

The composition of FAMEs was determined using a HP 6890 gas chromatograph (Agilent, California, USA) equipped with a flame ionization detector (FID) and DB-23 column (60 m × 0.32 mm × 0.25 μm). Nitrogen was used as the carrier gas at a flow rate of 1.5 mL min^−1^ with a split ratio of 1:30. The injector and detector temperatures were set at 250 °C and 280 °C, respectively. The oven temperature program started at 150 °C and increased to 210 °C at a rate of 5 °C min^−1^, followed by a holding period of 25 min at 210 °C. Biodiesel characteristics, including cetane number, iodine value, degree of unsaturation (DUS), kinematic viscosity, specific gravity, cloud point, higher heating value (HHV), long-chain saturated factor (LCSF), and cold filter plugging point (CFPP), were estimated as previously explained [37] and compared with the US ASTM biodiesel standard [38] as well as the European standard [39].

### 2.6. Statistical Analysis

All experiments were carried out in triplicate, and the results are presented as mean ± standard deviation. Statistical analysis was performed using the IBM SPSS Statistics software v.20 package. Differences among treatments were evaluated using analysis of variance (ANOVA), followed by the least significant difference (LSD) test at a significance level of *p* ≤ 0.05.

## 3. Results

### 3.1. Physicochemical Properties of Aquaculture Wastewater

The salinity of AWW (31.2 ± 1.6 ppt) was comparable to that recommended for f/2 medium (measured average salinity 30.4 ppt), indicating suitable saline conditions compared to the standard marine growth medium. This species is well known for its ability to thrive in saline environments ranging from 20 to over 35 ppt, with optimal growth frequently reported near seawater salinity (~30–35 ppt) [40]. The similar salinity between AWW and f/2 suggests that no major salinity adjustment would be required prior to cultivation in AWW. The pH values of AWW showed 7.61, and the recommended pH value for f/2 is 8.0 [29]. Maintaining near-neutral to slightly alkaline pH is critical for nutrient availability and photosynthetic efficiency in microalgal cultures [41]. Although the pH values of AWW and f/2 were very close and within the optimal range (7.0–8.5) reported for *D. salina* [42], the pH of the AWW was adjusted to 8.0 using 0.1 M sodium hydroxide to keep comparable conditions.

A marked difference between the AWW and f/2 medium was observed in nitrogen content and source (Table 1). While f/2 contained 75 mg L^−1^ nitrate in the form of NaNO_3_ as the sole nitrogen source, AWW exhibited a complex nitrogen profile, including nitrate (132.34 mg L^−1^), nitrite (23.92 mg L^−1^), and a substantially elevated ammonium concentration (421.62 mg L^−1^). The high ammonium concentration in AWW is typical of aquaculture effluents due to fish excretion and feed degradation [1,43]. Ammonium is generally considered a preferred nitrogen source for many microalgae because it can be assimilated directly without prior reduction, thereby saving cellular energy [44,45]. However, excessive ammonium may cause toxicity, particularly at elevated pH where the equilibrium shifts toward free ammonia (NH_3_) [46]. Given the adjusted pH 8.0, most nitrogen would remain in the ionized NH_4_^+^ form, potentially mitigating toxicity risks. In general, the presence of multiple nitrogen forms in AWW could promote rapid initial growth of *D. salina*, though careful monitoring would be required to avoid inhibitory effects. Additionally, the relatively high total nitrogen content in AWW compared with f/2 indicates strong potential for simultaneous nutrient removal and biomass production, supporting the dual function of phycoremediation and biomass generation [5,9].

Phosphate concentrations were similar between AWW (5.92 mg L^−1^) and f/2 (5.65 mg L^−1^), suggesting that phosphorus would not be a limiting nutrient in either medium. Adequate phosphorus is essential for nucleic acid synthesis and energy metabolism (ATP, NADPH), directly influencing the growth rate and productivity [41]. Sulfate levels were considerably higher in AWW (4.21 mg L^−1^) compared to f/2 (0.032 mg L^−1^). Sulfur is required for the synthesis of amino acids such as cysteine and methionine and may influence stress responses and metabolic activity in microalgae [47]. Elevated sulfate in AWW may thus contribute to enhanced metabolic activity without posing toxicity concerns at the measured concentration. Overall, the physicochemical profile of AWW demonstrates strong compatibility with the growth requirements of *D. salina*. Comparable salinity and pH to the standard f/2 medium, combined with substantially higher nitrogen availability, suggest that AWW can serve as a cost-effective alternative cultivation medium.

### 3.2. Growth Performance and Biomass Accumulation

**Figure 1:**
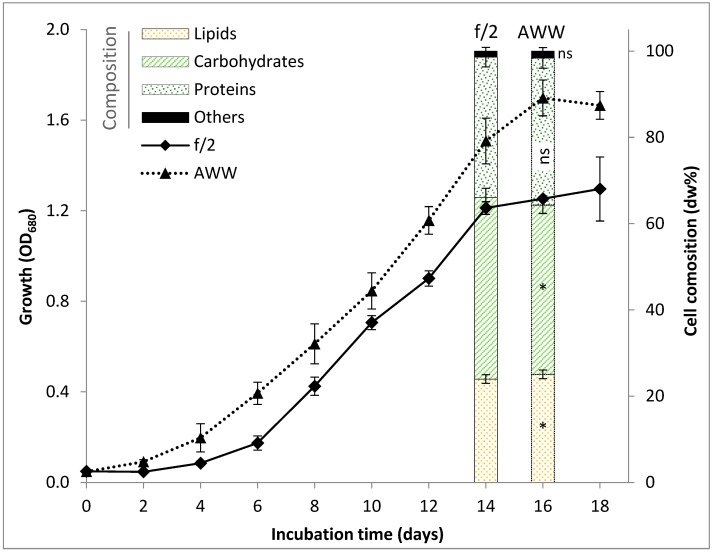
Growth performance of *Dunaliella salina* cultivated in synthetic f/2 medium and aquaculture wastewater (AWW) for 18 days, showing the biochemical composition at the late exponential phase. * and ns refer to significant and insignificant differences, respectively, with respect to the corresponding value of the same component in f/2 medium (at *p* ≤ 0.05).

Cultivation of *D. salina* in AWW resulted in consistently superior growth compared to the synthetic f/2 medium (Figure 1). AWW exhibited a short lag phase (<2 days), followed by exponential growth, while f/2 showed a ~4 day lag phase. However, AWW-supported cultures reached a higher optical density at the end of exponential phase (1.7 at day 16) than those grown in f/2 (1.2 at day 14), indicating enhanced biomass formation. This trend was confirmed by dry weight measurements (Table 2). Final biomass yield in AWW reached 1.320 g L^−1^, significantly 41% higher than that in f/2 (0.937 g L^−1^). Accordingly, biomass productivity increased from 0.076 g L^−1^ d^−1^ in f/2 to 0.090 g L^−1^ d^−1^ in AWW.

The improved performance in AWW can be primarily attributed to its nutrient composition rather than salinity effects. Although both media exhibited comparable salinity (~30–31 ppt), halophilic species such as *Dunaliella salina* are generally capable of growing efficiently at high salinities, where the energetic demand for osmotic regulation is lower than under extreme hypersaline conditions. Instead, the superior biomass accumulation in AWW is more convincingly explained by its higher total nutrient content and the presence of multiple nitrogen forms. Unlike f/2 medium, which supplies nitrogen solely as nitrate, AWW contains a mixture of ammonium, nitrate, and nitrite. Ammonium, in particular, can be directly assimilated into cellular metabolism without requiring reduction [48], thereby conserving energy and supporting faster growth. Furthermore, the elevated sulfate concentration in AWW may have contributed to enhanced synthesis of sulfur-containing amino acids and improved metabolic activity. The combined availability of diverse and readily assimilable nutrients in AWW likely played a dominant role in promoting biomass production, outweighing any minor effects associated with salinity under the tested conditions. Thus, the results suggest that nutrient composition, especially nitrogen availability and form, is a key factor driving the enhanced growth of *D. salina* in AWW. It is noteworthy to mention that a recent study showed 40–60 ppt optimal salinity range for this *D. salina* isolate [49]. This explains the slightly lower biomass yield obtained in the present study compared to hypersaline brine.

### 3.3. Biochemical Composition

Biochemical analysis revealed a balanced macromolecular composition in both media, with carbohydrates, proteins, and lipids constituting the major fractions (Table 2). Lipid content was significantly higher in AWW-grown biomass (250.6 mg g^−1^ dw) than in f/2 (239.8 mg g^−1^ dw). Protein content showed insignificant changes using AWW (340.9 mg g^−1^ dw) compared to f/2 (325.1 mg g^−1^ dw). In contrast, carbohydrate content was 6.8% significantly lower in AWW compared to f/2. The significant increase in lipid content suggests partial carbon reallocation toward storage compounds, a phenomenon frequently observed during late exponential growth in microalgae [31,50]. The reduction in carbohydrate proportion under AWW may, therefore, reflect redistribution of fixed carbon toward lipid accumulation.

Beyond cellular composition, volumetric productivities of key biomolecules were significantly enhanced in AWW (Table 2). Lipid productivity increased from 18.7 mg L^−1^ d^−1^ in f/2 to 23.6 mg L^−1^ d^−1^ in AWW. Carbohydrate productivity rose also from 32.9 to 37.0 mg L^−1^ d^−1^, while protein productivity increased from 25.4 to 32.1 mg L^−1^ d^−1^. These improvements are primarily driven by higher biomass productivity in AWW, together with the relatively slight differences in the intracellular biochemical composition. It is noteworthy to mention that growth could be further enhanced by increasing the salinity to the optimal range for this isolate. Overall, AWW improved the productivity of all major macromolecules through enhanced biomass yield, suggesting that it represents a promising cultivation medium for *D. salina*.

### 3.4. Desalination Performance and Phycoremediation

**Figure 2:**
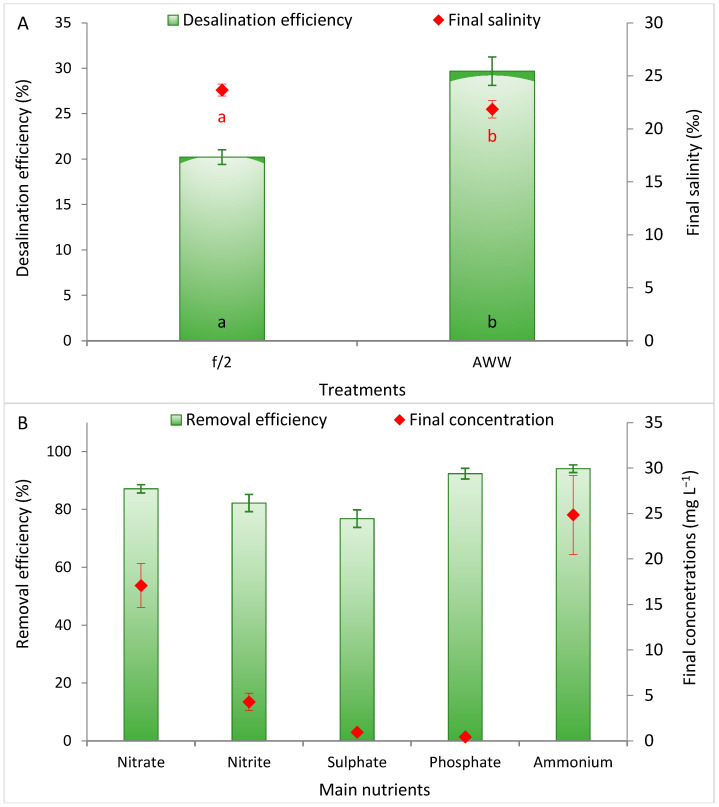
Desalination efficiency (%) showing the final salinity (ppt) (**A**) after cultivation of *Dunaliella salina* in f/2 medium and aquaculture wastewater (AWW) and removal efficiency of the main nutrients from AWW, showing the residual concentrations (**B**). Different letters in the same series refer to significant differences (at *p* ≤ 0.05).

Cultivation of *D. salina* resulted in measurable salinity reduction in both treatments (Figure 2A). The final salinity values decreased significantly in both treatments. In f/2 cultures, salinity declined to 23.7 ppt, whereas in AWW it decreased more markedly to 21.9 ppt. Therefore, desalination efficiency reached 20.2% in the f/2 medium and increased significantly to 29.7% in AWW (Figure 2A). The higher desalination efficiency observed in AWW may be attributed to greater biomass accumulation and metabolic activities. As a halophilic species, *D. salina* maintains osmotic balance primarily through glycerol synthesis and active ion transport mechanisms [13,42]. These physiological adaptations can contribute to net salt removal from the surrounding medium.

In addition to salinity reduction, *D. salina* cultivation in AWW showed strong phycoremediation performance, as illustrated by the high removal efficiencies of major nutrients (Figure 2B). Nitrate, nitrite, sulfate, phosphate, and ammonium removal efficiencies reached high levels, indicating effective assimilation of dissolved nutrients by the microalgal cells. Nutrient removal by microalgae occurs primarily through cellular uptake for biomass synthesis, assimilation into proteins and nucleic acids, and adsorption onto cell surfaces [51,52]. The substantial reductions in nutrient concentrations observed in AWW confirm the capability of *D. salina* to function as a biological treatment agent for aquaculture effluents. In this context, phycoremediation using microalgae has been widely recognized as a sustainable approach for wastewater treatment because it simultaneously removes nutrients, reduces environmental pollution, and generates valuable biomass for downstream applications such as biofuels and bioproducts [53,54]. Moreover, integrating microalgae into aquaculture systems has been proposed as an effective strategy to close nutrient loops, where microalgae can utilize waste nutrients while simultaneously providing oxygen and capturing carbon dioxide, thereby improving system sustainability and resource efficiency [1]. The high nutrient removal efficiencies observed in this study therefore highlight the dual role of *D. salina* cultivation in AWW by environmental remediation and biomass generation. Overall, these results demonstrate that *D. salina* cultivation can effectively integrate desalination and phycoremediation processes, supporting the potential use of aquaculture wastewater as a resource for sustainable microalgal production systems.

### 3.5. Total FAMEs and Productivity

The total FAMEs content of *D. salina* was slightly higher in AWW-grown cultures (235.69 mg g^−1^ dw) compared to f/2 (223.73 mg g^−1^ dw), representing an increase by only 5.3% (Table 3). Interestingly, volumetric FAMEs productivity increased substantially by 27.2% using AWW, reflecting the combined effect of higher biomass productivity and enhanced lipid accumulation in wastewater-grown cultures. FAMEs accounted for 93.3% and 94.0% of total lipids in f/2 and AWW, respectively, indicating that the extracted lipids were predominantly saponifiable fatty acids, which is favorable for biodiesel applications. Similar high FAMEs proportions have been reported for several microalgal species used as biodiesel feedstocks [17,55].

Palmitic acid (C16:0) was the dominant fatty acid in both media, contributing 30.8% of total FAMEs in f/2 and increasing to 36.7% in AWW (Table 3). Similarly, stearic acid (C18:0) and myristic acid (C14:0) were present at higher proportions in AWW-grown cells. The elevated saturated fatty acids (SFAs) under AWW conditions resulted in a marked increase in total SFAs content from 34.5% in f/2 to 44.0% in AWW. Monounsaturated fatty acids (MUFAs), including C16:1 and C18:1 isomers, also increased in AWW cultures (11.3%) compared to f/2 (7.2%). Oleic acid isomers (C18:1 n-9) were notably higher under AWW conditions, which is advantageous for biodiesel quality due to their positive influence on oxidative stability and cold flow properties [56]. In contrast, polyunsaturated fatty acids (PUFAs) decreased from 58.3% in f/2 to 44.7% in AWW. Linolenic acid (C18:3 n-3), a major PUFA, declined from 30.2% to 23.4%, and linoleic acid isomers (C18:2 n-6) also showed reduced proportions in AWW-grown biomass. The shift toward higher SFAs and MUFAs fractions and lower PUFAs content in AWW-grown cells indicates a reduction in overall unsaturation degree. From a biofuel perspective, this compositional shift is favorable, as high PUFAs content can negatively affect oxidative stability [57], as discussed in details in the next section. It is reported that nitrogen availability can influence the activity of desaturase enzymes, thereby altering the balance between saturated and unsaturated fatty acids [50]. Therefore, the observed changes in fatty acid distribution may be attributed to nutrient composition and environmental conditions in AWW, particularly high nitrogen and possible variations in micronutrients.

### 3.6. Fuel Property Evaluation

The predicted biodiesel properties derived from FAMEs profiles of *D. salina* grown in both media were within internationally accepted limits (Table 4), confirming the suitability of the produced lipids for biodiesel applications. It is noteworthy to mention that the predicted fuel properties serve as preliminary indicators of biodiesel quality and that further experimental validation, including engine testing, is required to confirm practical applicability. DUS decreased from 1.56 in f/2 to 1.26 in AWW, reflecting the reduction in polyunsaturated fatty acids observed under AWW cultivation. This shift is consistent with the increased SFAs and MUFAs fractions previously discussed. Lower DUS generally enhances oxidative stability, an important parameter for biodiesel storage [58]. Kinematic viscosity values were 4.22 mm^2^ s^−1^ in f/2 and 4.41 mm^2^ s^−1^ in AWW, both within the acceptable ranges specified by ASTM International D6751 (1.9–6.0 mm^2^ s^−1^) and European Committee for Standardization EN 14214 (3.5–5.0 mm^2^ s^−1^). These values indicate appropriate fuel flow characteristics for diesel engines. In addition, specific gravity remained constant at 0.88 kg L^−1^ in both treatments, falling within the ASTM recommended range (0.85–0.90 kg L^−1^). Proper density ensures optimal fuel injection and combustion efficiency [56]. Cloud point differed notably between treatments, with f/2-derived biodiesel showing a lower value (−0.84 °C) compared to AWW (3.17 °C). The higher cloud point in AWW-derived biodiesel is attributable to increased SFAs content, which tends to crystallize at higher temperatures [58]. Cetane number, an indicator of ignition quality, improved from 52.47 in f/2 to 54.48 in AWW. Both exceed the ASTM minimum requirement (47) and satisfy the European standard (≥51). The higher CN in AWW-derived biodiesel is consistent with its higher SFAs and MUFAs content, which generally increases ignition quality and combustion efficiency [56].

The iodine value, which reflects total unsaturation, decreased markedly from 128.75 g I_2_/100 g oil in f/2 to 106.37 g I_2_/100 g oil in AWW. The AWW-derived biodiesel meets the European EN 14214 maximum limit (120 g I_2_/100 g oil), whereas the f/2-derived biodiesel slightly exceeds this threshold. This further confirms that cultivation in AWW shifts fatty acid composition toward improved oxidative stability. The C18:3 content (linolenic acid) remained below the maximum limit of 12 wt% specified by both ASTM and EN standards (7.2% in f/2 and 5.9% in AWW). Additionally, fatty acids with four or more double bonds (Db ≥ 4) were absent in both treatments, well below the European limit (≤1%), indicating favorable stability characteristics. The higher heating values were comparable between treatments (41.28 MJ kg^−1^ for f/2 and 40.75 MJ kg^−1^ for AWW), and similar to values reported for other microalgal biodiesels (Table 4). Cold flow properties were further evaluated through both CFPP and LCSF. The cloud point increased from −0.84 °C in f/2 to 3.17 °C in AWW, which indicates earlier crystal formation due to higher SFAs content in AWW. However, the CFPP showed a contrasting trend, improving significantly from −0.07 °C in f/2 to −16.31 °C in AWW. This suggests enhanced low-temperature operability of AWW-derived biodiesel, likely influenced by the overall fatty acid distribution and lower LCSF, which decreased markedly from 5.22 to 0.05. Since CFPP is a more critical parameter than cloud point for cold-weather engine performance, these results indicate that AWW cultivation may improve fuel usability under low-temperature conditions.

Compared with other microalgae such as *Tetradesmus obliquus* and *Scenedesmus obliquus*, *D. salina* biodiesel exhibits competitive fuel properties. For instance, cetane number of *D. salina* biodiesel exceeded those reported for *T. obliquus* (50.76) and *S. obliquus* (51.5–51.6), as shown in Table 4. Furthermore, the iodine value of AWW-derived *D. salina* biodiesel was substantially lower than those reported for *T. obliquus* (147.89 g I_2_/100 g oil) and *S. obliquus* (≈138–140 g I_2_/100 g oil). DUS also decreased to 1.26 in AWW-grown *D. salina*, compared with higher values reported for *T. obliquus* (1.82) and *S. obliquus* (1.69–1.71), suggesting improved oxidative stability and better compliance with European biodiesel specifications. The kinematic viscosity values of *D. salina* biodiesel are comparable to those reported for *T. obliquus* and *S. obliquus*. Specific gravity remained constant at 0.88 kg L^−1^ across all microalgae, indicating similar fuel density and injection characteristics. Overall, these comparisons highlight that *D. salina* cultivated in AWW can produce biodiesel with fuel properties comparable to or superior to several commonly studied microalgal feedstocks, supporting its viability as an alternative and sustainable biodiesel source.

The present study showed that the enhanced desalination efficiency in AWW did not compromise lipid accumulation or fatty acid quality (see Table 3). On the contrary, AWW-supported cultures exhibited improved FAMEs productivity and a more favorable fatty acid profile for biodiesel applications. This indicates that salt removal and biofuel-oriented biomass production can be achieved concurrently. Collectively, the growth kinetics, biomass yield, biochemical composition, and productivities demonstrate that AWW not only substitutes synthetic f/2 medium but can surpass it under the tested conditions. The comparable salinity and pH, together with high nitrogen availability, provide favorable conditions for *D. salina* cultivation. These findings support the feasibility of coupling aquaculture operations with microalgal biotechnology as part of a circular bioeconomy strategy, reducing reliance on commercial nutrients while mitigating environmental discharge impacts. Despite these promising results, several important biodiesel quality parameters, including oxidative stability, flash point, glycerin content, saponification value, and acid number, were not determined in this study. These parameters are critical for comprehensive fuel assessment and regulatory compliance. Therefore, future work should include detailed experimental evaluation of biodiesel properties to validate the suitability of *D. salina*-derived biodiesel for commercial applications.

## 4. Conclusions

The present study demonstrates the feasibility of cultivating *D. salina* in AWW as a sustainable alternative to synthetic f/2 medium. The physicochemical characteristics of AWW, particularly its salinity, pH, and high nitrogen availability, supported efficient microalgal growth without major adjustments. Cultivation in AWW significantly enhanced biomass production, reaching 1.320 g L^−1^ with a productivity of 0.090 g L^−1^ d^−1^, approximately 41% higher than that obtained in f/2 medium. Consequently, volumetric productivities of lipids, proteins, and carbohydrates were also improved. In addition to biomass generation, *D. salina* cultivation contributed to environmental remediation. Salinity decreased by 29.7% in AWW, demonstrating the potential of this halophilic species to contribute to partial desalination during cultivation. High removal efficiencies of nitrate, nitrite, ammonium, phosphate, and sulfate confirmed the strong phycoremediation capacity of the microalga. The lipid fraction was suitable for biodiesel production, with FAMEs accounting for 94% of total lipids. Cultivation in AWW improved FAMEs productivity and shifted fatty acid composition toward higher SFAs and MUFAs, resulting in favorable biodiesel properties that comply with international standards. Overall, integrating *D. salina* cultivation with AWW treatment offers a promising approach for simultaneous wastewater remediation and sustainable biofuel-oriented biomass production. Future research should focus on validating the present findings under pilot- and large-scale cultivation systems to assess process stability and economic feasibility under real operational conditions. In addition, experimental evaluation of biodiesel properties, including oxidative stability, flash point, acid value, and glycerol content, is necessary to complement the theoretical predictions reported in this study. Moreover, exploring the potential of the produced biomass for additional value-added products within a biorefinery framework may further improve the overall economic viability of the process.

## Figures and Tables

**Table 1:** Physicochemical characteristics of the synthetic f/2 medium and aquaculture wastewater (AWW) used for cultivation of *Dunaliella salina*.

Parameters	f/2 *	AWW
Salinity (ppt)	30.4	31.2 ± 1.6
pH	8.0	7.61 ± 0.49
Temperature (°C)	*ni*	26.3 ± 1.2
Ions (mg L^−1^)		
Nitrate	75 ^#^	132.34 ± 4.06
Nitrite	*ni*	23.92 ± 1.49
Sulfate	0.032 ^#^	4.21 ± 0.75
Phosphate	5.65	5.92 ± 0.32
Ammonium	*ni*	421.62 ± 28.18

* Calculated from the medium composition, *ni* not included in the medium. ^#^ as NaNO_3_ and as the sum of CuSO_4_.5H_2_O and ZnSO_4_.7H_2_O, respectively.

**Table 2:** Biomass concentration, biochemical composition, and productivities of *Dunaliella salina* cultivated in synthetic f/2 medium and aquaculture wastewater (AWW).

Parameters		f/2	AWW
Dry weight (g L^−1^)		0.937 ± 0.022	1.320 ± 0.062 *
Contents (mg g^−1^ dw)	Lipids	239.8 ± 8.5	250.6 ± 3.8 *
	Carbohydrates	421.1 ± 11.4	392.6 ± 16.6 *
	Proteins	325.1 ± 7.8	340.9 ± 12.6 ^ns^
Productivities	Biomass (g L^−1^ d^−1^)	0.076 ± 0.002	0.090 ± 0.005 *
	Lipids (mg L^−1^ d^−1^)	18.7 ± 0.66	23.6 ± 0.36 *
	Carbohydrates (mg L^−1^ d^−1^)	32.9 ± 0.89	37.0 ± 1.57 *
	Proteins (mg L^−1^ d^−1^)	25.4 ± 0.61	32.1 ± 1.19 *

* and ^ns^ refer to significant and insignificant differences, respectively, with respect to the corresponding value of f/2 medium (at *p* ≤ 0.05). Biomass productivity was calculated based on the dry weight at early exponential phase (day 2 for AWW and day 4 for f/2) and late exponential phase (day 16 for AWW and day 14 for f/2).

**Table 3:** Fatty acid methyl ester (FAME, mg g^−1^ dw) profile, distribution groups, and FAME productivity of *Dunaliella salina* cultivated in synthetic f/2 medium and aquaculture wastewater (AWW).

Fatty Acids	f/2	AWW
14:0	0.64 ± 0.15 (0.3)	2.68 ± 0.24 (1.1)
16:0	68.82 ± 2.67 (30.8)	86.56 ± 0.71 (36.7)
16:1	7.26 ± 0.23 (3.2)	10.67 ± 0.39 (4.5)
18:0	5.61 ± 0.51 (2.5)	11.20 ± 0.25 (4.8)
18:1(n-9)t	4.13 ± 0.16 (1.8)	7.47 ± 0.12 (3.2)
18:1(n-9)c	4.78 ± 0.20 (2.1)	8.43 ± 0.89 (3.6)
18:2(n-6)t	48.76 ± 0.98 (21.8)	39.18 ± 1.53 (16.6)
18:2(n-6)c	7.51 ± 0.49 (3.4)	4.50 ± 0.51 (1.9)
18:3(n-3)	67.47 ± 1.84 (30.2)	55.11 ± 1.33 (23.4)
18:3(n-6)	4.43 ± 0.32 (2.0)	4.35 ± 0.45 (1.8)
20:0	2.00 ± 0.22 (0.9)	3.28 ± 0.35 (1.4)
20:2	2.32 ± 0.20 (1.0)	2.26 ± 0.30 (1.0)
SFAs	77.07 ± 2.81 (34.5)	103.73 ± 0.25 * (44.0)
MUFAs	16.16 ± 0.55 (7.2)	26.57 ± 1.40 * (11.3)
PUFAs	130.50 ± 1.85 (58.3)	105.39 ± 2.21 * (44.7)
Total FAMEs (mg g^−1^ dw)	223.73 ± 3.57 (100.0)	235.69 ± 0.79 * (100.0)
FAMEs productivity (mg L^−1^ d^−1^)	17.47 ± 0.28 (na)	22.22 ± 0.07 * (na)
FAMEs (% of total lipids)	93.30 ± 1.49 (na)	94.05 ± 0.32 ^ns^ (na)

Values in brackets indicate the percentage contribution of each FAME relative to the total identified FAMEs. * and ^ns^ refer to significant and insignificant differences, respectively, with respect to the corresponding value of f/2 medium (at *p* ≤ 0.05). na: not available.

**Table 4:** Estimated biodiesel properties of fatty acid methyl esters (FAMEs) derived from *Dunaliella salina* cultivated in f/2 medium and aquaculture wastewater (AWW), compared with other microalgae and international standards.

Characteristics	f/2	AWW	Microalgae			International Standards	
			T.o.	S.o.	S.o.	US	Europe
DUS	1.56	1.26	1.82	1.69	1.71	-	-
Kinematic viscosity (mm^2^ s^−1^)	4.22	4.41	4.06	4.14	4.13	1.9–6.0	3.5–5.0
Specific gravity (kg^−1^)	0.88	0.88	0.88	0.88	0.88	0.85–0.9	-
Cloud point (°C)	−0.84	3.17	−4.28	−2.59	−2.78	-	-
Cetane number	52.47	54.48	50.76	51.60	51.50	Min. 47	51–120
Iodine value (g I_2_/100 g oil)	128.75	106.37	147.89	138.45	139.60	-	Max. 120
HHV (MJ kg^−1^)	41.28	40.75	41.73	41.51	41.50	-	-
C18:3 (wt %)	7.2	5.9	*na*	*na*	*na*	Max. 12	Max. 12
Db ≥ 4 (wt %)	0.00	0.00	*na*	3.14	3.54	≤1	-
LCSF (wt %)	5.22	0.05	0.209	*na*	3.9	-	-
CFPP (°C)	−0.07	−16.31	−15.82	*na*	−4.4	-	≤5/≤−20
References	This study		[57]	[30]	[59]	[38]	[39]

DUS refers to degree of unsaturation, LCSF and CFPP refer to long-chain saturated factor and cold filter plugging point, respectively. T.o. and S.o. refer to *Tetradesmus obliquus* and *Scenedesmus obliquus*, respectively. *na* not available.

## Data Availability

The data supporting the findings of this study are included in this article.
